# Dengue virus infection – a review of pathogenesis, vaccines, diagnosis and therapy

**DOI:** 10.1016/j.virusres.2022.199018

**Published:** 2022-12-07

**Authors:** Boon Hui Kok, Hui Ting Lim, Chin Peng Lim, Ngit Shin Lai, Chiuan Yee Leow, Chiuan Herng Leow

**Affiliations:** a Institute for Research in Molecular Medicine (INFORMM), Universiti Sains Malaysia, Gelugor, Penang 11800, Malaysia; b School of Pharmaceutical Sciences, Universiti Sains Malaysia, Gelugor, Penang 11800, Malaysia

**Keywords:** DENV, Cross reaction, Development, Therapeutic, Diagnostic

## Abstract

The transmission of dengue virus (DENV) from an infected *Aedes* mosquito to a human, causes illness ranging from mild dengue fever to fatal dengue shock syndrome. The similar conserved structure and sequence among distinct DENV serotypes or different flaviviruses has resulted in the occurrence of cross reaction followed by antibody-dependent enhancement (ADE). Thus far, the vaccine which can provide effective protection against infection by different DENV serotypes remains the biggest hurdle to overcome. Therefore, deep investigation is crucial for the potent and effective therapeutic drugs development. In addition, the cross-reactivity of flaviviruses that leads to false diagnosis in clinical settings could result to delay proper intervention management. Thus, the accurate diagnostic with high specificity and sensitivity is highly required to provide prompt diagnosis in respect to render early treatment for DENV infected individuals. In this review, the recent development of neutralizing antibodies, antiviral agents, and vaccine candidates in therapeutic platform for DENV infection will be discussed. Moreover, the discovery of antigenic cryptic epitopes, principle of molecular mimicry, and application of single-chain or single-domain antibodies towards DENV will also be presented.

## Highlights

• Reviews on unsolved issues of DENV from different perspectives such as the effect of antibody dependent enhancement, immune escape and limitations of commercial diagnostic kits.
• Case studies on variety of potential therapeutic candidates targeting on DENV such as peptide inhibitors, broadly neutralizing antibodies, engineered antibody, vaccine candidates and antiviral agents.
• Exploring different approaches for the improvisation of DENV diagnosis such as application of bio-functionalized tapered optical fiber, nanobody derivatives and antibody engineering.

## 1 Introduction

**Fig. 1:**
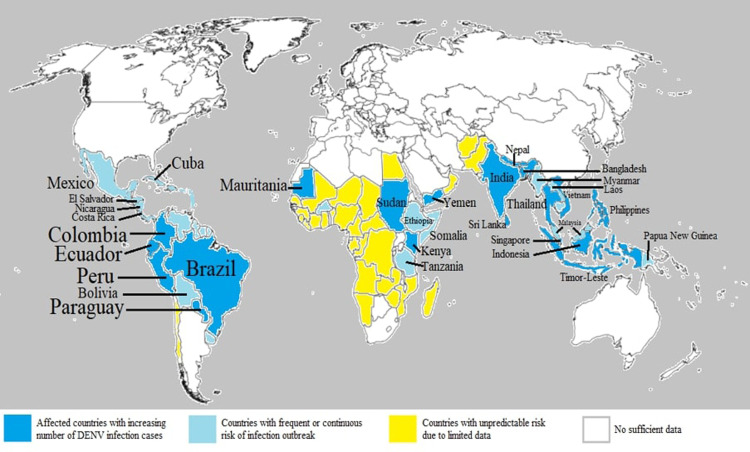
Global dengue epidemic in 2020 and 2021. The affected countries include Bangladesh, Philippines, Vietnam, India, Maldives, Indonesia, Singapore, Colombia, Paraguay, Peru, Kenya, Fiji, Cook Islands, Reunion Islands, Sri Lanka, Thailand, Sudan, Mauritania, Timor-Leste, Yemen, Nepal, Mayotte, Ecuador and Brazil. Info adapted from (CDC, 2021; ECDC, 2021; WHO, 2021).

Dengue virus infection is one of the most common mosquito-borne diseases occurring in both tropical and subtropical regions which causes up to about 100 to 400 million infected cases per year globally (WHO, 2021). Currently, DENV infection is endemic to countries such as Africa, Eastern Mediterranean, the Americas, Southeast Asia and Western Pacific (Chaturvedi and Shrivastava, 2004). The distribution of the global dengue epidemic indicates dengue infection outbreak is happening all over the world (Fig. 1). According to recent WHO report, the increasing number of infected cases in 2020 has made DENV a serious virus caused disease after COVID-19 (WHO, 2021). Of these countries, Philippines, Vietnam, India, Colombia and Brazil were reported to have the highest DENV infected cases (ECDC, 2021). One of the main factors which causes the dissemination of mosquitoes-borne diseases in global is the speedy urbanization with improper infrastructure planning that could lead to inefficient vector control management. Besides, business troupe or personal travel also facilitate the spreading of these mosquitoes-borne diseases into new environments when they travel from one place to another place (Kraemer et al., 2015).

In 2013, a newly discovered serotype of DENV was reportedly found in Sarawak, Malaysia. Initially, this DENV case was considered as the sylvatic dengue infection from DENV4 which involves the transmission between mosquitoes *Aedes nivalis* and non-human primates (Normile, 2013). After a series of genetic verification process, it was identified as new DENV5 serotype and predominantly happened in the forest of South-East Asia (Mustafa et al., 2015). Despite the actual causative transmission of DENV5 remains unclear, the possible factors that lead to the emergence could be due to the genetic changes from sylvatic strains to human strains (Vasilakis et al., 2011), high mutation frequency of DENV (Monath, 1994) and massive deforestation (Rudel et al., 2009). This new DENV5 serotype which shows distinct phylogenetic from the other 4 existing serotypes (Mustafa et al., 2015; Taylor-Robinson, 2016) indicates that new emergence of DENV is ongoing, foretelling that there will be more arising virus emergence happening possibly due to the zoonotic transmission, owing to changes in climate and ecosystem (Joob and Wiwanikit, 2018).

So far, the specific therapeutic drugs or vaccine for DENV infection is still unavailable. One of the reasons is the cross reactivity usually occurred between DENV and the other flaviviruses due to the similar conserved structure presented on the envelope protein (E) shared among the flaviviruses. As reported earlier, DENV E protein exhibited up to 50% of the same homology with ZIKV E protein (Sirohi et al., 2016), hence leading to the occurrence of cross reactivity followed by ADE during the second infection from any members of the flaviviruses based on the specificity and amount of cross reactive antibodies generated by the immune system (Montecillo-Aguado et al., 2019). Besides, the co-infections between different flaviviruses such as zika virus (ZIKV) and chikungunya virus (CHIKV) or different DENV serotypes have always presented similar onset symptoms thereby it could lead to the complication for clinical diagnosis management (Musso et al., 2015). To address this problem, the accurate and rapid diagnosis tool with high specificity and sensitivity is crucial for early medication treatment (Peeling et al., 2010).

In this review, a few focuses on the DENV infection issues such as cross reactivity due to different serotypic DENV infection and different flavivirus infection, ADE, immune evasion and limitations of commercial diagnostic kits will be overviewed. Besides, recent diagnostic and therapeutic developments with different approaches will be reviewed and presented to suggest possible ways on resolving the issue of cross reaction. Indeed, the discovery of potential therapeutic agents and diagnostic markers will never come to an end until the generation of specific, sensitive diagnostic marker and vaccines or therapeutic agents which are sensitive and effective in treating DENV infection.

### 1.1 Dengue virus genome and structure

**Fig. 2:**
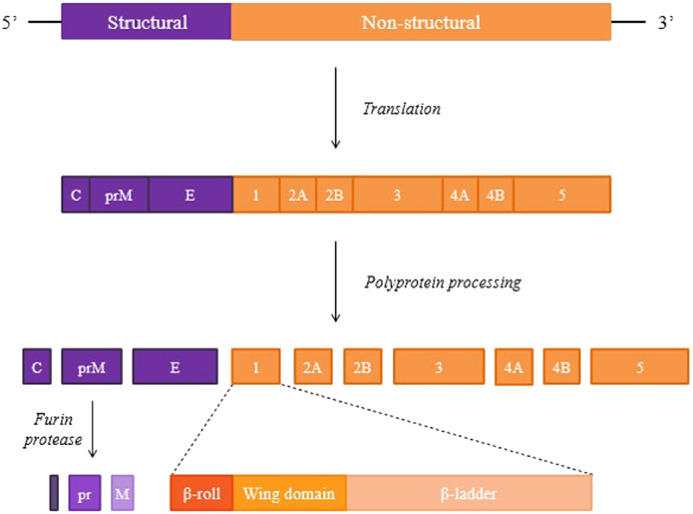
Diagram of DENV genome and NS1 protein domains. Viral genome undergoes translation to generate single polyprotein. After the polyprotein processing, viral proteins are produced via proteolytic cleavages (Fernandez-Garcia et al., 2009). For NS1, the 3 domain regions involving in DENV pathogenesis include β-roll, wing domain and β-ladder (Scaturro et al., 2015).

DENV is one of the Flavivirus that derived from Flaviviridae family (Murugesan and Manoharan, 2020). Generally, critical symptoms such as dengue shock syndrome (DSS) and dengue haemorrhagic fever (DHF) could be generated by each consecutive infections from any of the four antigenically distinct DENV serotypes (DENV-1, DENV-2, DENV-3, DENV-4) (Morens, 1994). DENV is a small icosahedral enveloped virus (Rey et al., 2018) consists of 11 kb positive single stranded RNA (Clyde and Harris, 2006) which enclosed three types of structural proteins including capsid (C), envelope (E) and membrane (M) and seven non-structural (NS) proteins such as NS1, NS2A, NS2B, NS3, NS4A, NS4B and NS5 within its matured enveloped structure known as virions (Tremblay et al., 2019) (Fig. 2). The structural proteins mainly involved in virus assemble. For example, C protein interacts with RNA to assemble nucleocapsid (Ma et al., 2004). The formation of mature virus particle in E protein requires the help of M protein which is important in recognizing different types of immune responses against different flaviviruses infection (Cardosa et al., 2002). It is also the major components in DENV which acts as a surface protein in assisting the virus attachment and fusion on host cell membrane (Modis et al., 2004). Other than that, M protein can form into structural virus particle through oligomerization (Wong et al., 2012). On the other hand, non-structural proteins and cellular proteins are essentials for viral genome's replication, translation, encapsidation and proper folding of viral proteins which all happen in the cytoplasm associated with rough endoplasmic reticulum (Clyde and Harris, 2006).

### 1.2 Life cycle of DENV

Human normally gets infected by dengue virus (DENV) through infected mosquitoes such as *Aedes aegypti* or *Aedes albopictus* (Bhatt et al., 2013). DENV will target on the dendritic cells (DCs) and macrophages during first day of infection (Kyle et al., 2007). To summarized from other reviews (Campos et al., 2018; Kato and Hishiki, 2016), DENV life cycle involves processes such as viral entry and attachment, virus and endosome membrane fusion, nucleocapsid release, protein synthesis and processing, RNA replication, nucleocapsid formation, viral assembly, viral maturation and finally the releasing of matured DENV particle. For the host cell attachment, DENV E protein will interact with cellular factors of the target cell such as dendritic cell-specific intercellular adhesion molecule-3-grabbing non-integrin (DC-SIGN), mannose receptor, heparan sulfate and others (Cruz-Oliveira et al., 2015). Next, DENV enters the targeted cell by clathrin-mediated endocytosis and the low pH within the endosomal compartment (Kato and Hishiki, 2016) allows the fusion between DENV and the endosome membrane. With the aid of acidic endosomal environment, RNA genome is released from the uncoating nucleocapsid into the cytoplasm to undergo protein processing and replication (Campos et al., 2018). Initially, the RNA genome act as mRNA and undergo translation to produce viral protein.

The replication of RNA genome occurs in the virus-induced intracellular membrane known as replication complex which comprised of viral RNA, viral proteins and host cell factors (Teo and Chu, 2014; Welsch et al., 2009). This complex which formed on the endoplasmic reticulum (ER) membrane also helps to protect the replication products from the recognition of host innate immune system (Pierson and Diamond, 2020). After the formation of nucleocapsid, DENV particle starts to assemble when it buds into the lumen of ER as immature DENV particle (Campos et al., 2018). Along the secretion pathway within the trans-Golgi network (TGN), the maturation of DENV requires the cleavage of pre-membrane/membrane (prM/M) to M protein by the action of furin-like serine proteases to release the DENV in its matured and infectious form (Elshuber et al., 2003). To avoid the premature DENV membrane from fusing with the host cell before the viral particle gets to release from the infected cell, the pr will enclose the hydrophobic fusion loop by continue binding with the E dimers after cleavage. Lastly, the pr will eventually detach from the surface of viral particles once the matured DENV is released into the extracellular space (Perera and Kuhn, 2008; Yu et al., 2008).

### 1.3 Clinical manifestation

Dengue infection involves the febrile phase, critical phase and recovery phase. Febrile phase normally happens for a week with symptoms such as high fever, flu, headaches, vomiting and joint pain. Critical phase also refers to life-threatening phase with the occurrence of more acute symptoms such as plasma leakage and internal bleeding. At the stage of recovery phase, symptoms become milder with the recovery of vascular permeability (Simmons et al., 2012).

DENV infection can cause a wide range of symptoms. During the incubation period from Day 4 to 10 (WHO, 2009), the disease may be asymptomatic or a mild acute febrile illness known as dengue fever (DF) while critical phase usually begins from Day 3 to 7 (WHO, 2021). Dengue haemorrhagic fever (DHF) comes with unusual vascular permeability which might further leads to sudden hypovolemic shock known as dengue shock syndrome (DSS), a critical phase for infected patient with severe dengue (Halstead, 1980).

Infection of DENV can lead to different level of seriousness depending on the serotypes, genetic variations and viral virulence (Martina et al., 2009). Different dengue serotypes may have different impact on the pathogenesis due to their structural differences (Leitmeyer et al., 1999) and viral replication rates (Vaughn et al., 2000). Besides, the changes in nucleotides such as amino acid mutations or substitutions will affect the viral virulence as the variations in nucleotides will eventually leads to different viral-host interactions and dissemination capability (Zou et al., 2019).

## 2 Reviews on unsolved issues of DENV

In this section, the immune response involving innate and adaptive immune response are briefly reviewed. We also reviewed on some unsolved issues such as the immune evasion of DENV, cross reaction within DENV serotypes, cross reaction among different flaviviruses, different antibody dependence enhancement (ADE) mechanisms and its effects. The limitations of current diagnostic kits will be discussed in Section 4.2.

### 2.1 Immune response towards DENV infection

The dendritic cells (DCs) are the connectors between innate and adaptive immune responses during invasion of viral particles (Steinman and Banchereau, 2007). It is in-charge of the upregulation of pro-inflammatory cytokines and co-stimulatory molecules which helps to activate the human immune responses (Ho et al., 2001; Libraty et al., 2001). Hence, DCs will be unable to stimulate the secretion of IFN-γ from Th1 cells when its maturation and migration are interfered by NS1 through regulation of related gene expression (Fernandez-Sesma et al., 2006). In order to ensure inactivation of influenza virus NS1, the antiviral agent must be capable to restore the host antiviral responses such as the innate immunity related to IFN production and restrict the virus replication (Yodsheewan et al., 2013).

DCs will extend the target antigen to the T cells such as CD8^+^ and CD4^+^ as the starting point of innate immune response in the draining lymph nodes (LNs) (St John and Rathore, 2019). The activated skin mast cell plays a crucial role in activating the recruitment of cytotoxic cells like CD8^+^ T cells, natural killer (NK) cells and natural killer T (NKT) cells to the infection sites in order to stimulate the virus termination and at the same time controlling the infection in the draining LNs (St John et al., 2011). CD8^+^T cells can terminate the infected cells directly by targeting on a variation of dengue proteins such as NS3 with the help of activated CD4^+^ T cells (Mathew et al., 1996). CD8^+^ T cells usually focused on non-structural proteins while CD4^+^ T cells are more towards the capsid, envelope and NS1 epitopes (Simon-Lorière et al., 2017). The roles of CD4^+^ T cells include helping B cell response, assisting in extracting memory responses from CD8 T cells (Mapalagamage et al., 2022; Swain et al., 2012) and providing cytotoxic effect (Rivino, 2018) and many other functions of the specialised CD4^+^ T cells as reviewed by Mapalagamage and her team (Mapalagamage et al., 2022).

The activation of complement system is important to block the virus infection in early stage of innate immune response. However, immune evasion of DENV can be facilitated by NS1 when it interacts with the respective complement components in different complement activation pathways such as classical and lectin pathways (Avirutnan et al., 2008). This will result in altering the functionality of complement components and inhibit complement-mediated response (Avirutnan et al., 2011; Kraivong et al., 2021; Thiemmeca et al., 2016). Through these strategies, DENV increases its chances of survival and viral replication. Besides, the interaction between NS1 and NS4B will regulate the RNA replication by co-localizing with double stranded RNA (Youn et al., 2012).

The viral replication of DENV in the early stage is normally controlled by the production of interferons (IFNs) which is known as the first line of defense (Rodenhuis-Zybert et al., 2010). The presence of DENV will be recognized by the toll-like receptors (TLRs) and C-type lectin receptors which are categorized as the pattern recognition receptors (PRRs) produced from immune sentinels (St John and Rathore, 2019) once the virus enters the human skin. The activation of PRRs activates the antiviral innate responses in human body through the production of IFNs and tumor-necrosis factor (TNF) (Ho et al., 2001). The activation of TLR-3 and TLR-7 will lead to the production of IFN-α and IFN-β which are important for the inhibition of DENV infection. The binding reaction between the IFNs and IFN receptors will trigger the Janus kinase-signal transducer and activator of transcription (JAK-STAT) pathway which further enhances the production of effector proteins up to 100 units (Ho et al., 2005).

Other than IFNs, intracellular sensors including retinoic acid-inducible gene 1 (RIG-I) and helicases melanoma differentiation-associated protein 5 (MDA5) are also one of the first line defense responsible for the viral RNA recognition and they also help in the production of IFN-β (Guzman and Harris, 2015). The activated RIG-I will be transferred to the mitochondria to interact with mitochondrial antiviral-signaling protein (MAVS) after the detection of viral RNA. The interaction between RIG-I and MAVS will further leads to the aggregation of MAVS to act as the immune signalosome which initiates the nuclear factor kB (NF-κB) and transcription factor IRF3 to induce the production of type I IFN after the translocation of the transcription factors into the nucleus (Liu et al., 2012).

### 2.2 Evasion of DENV from immune system

The evasion of DENV from immune system normally occurs due to the inhibition of immune response activation pathway. For example, DENV managed to bypass human's first line of defense by inhibiting the MAVS pathway. One of the strategies is to block the RIG-I from moving into the mitochondria with the interruption of NS3 protein (Muñoz-Jordán et al., 2005). Besides than NS3, NS4A also able to inhibit the binding between RIG-I and MAVS by binding to the MAVS caspase activation and recruitment domains (CARDs) (He et al., 2016). Another way of viral escape from the host pathogen recognition receptor is by interfering with the activation of TLR-3 antiviral signal (Morrison and Scholle, 2014).

On the other hand, DENV also able to inhibit the interference RNA (RNAi) pathway which is an important pathway in generating innate antiviral response. The sub-genomic flavivirus RNA (sfRNA) will be produced from 3′-untranslated region of the viral RNA to prevent the Dicer enzyme from cleaving the double stranded RNA (Schnettler et al., 2012). With the modulation of the host RNAi/microRNAs (Baneyx and Mujacic, 2004) pathway by the expression of NS4B, the RNAi pathway will be interfered and ease the replication of DENV (Kakumani et al., 2013). NS5 can affect the production of IFN by blocking the recruitment of transcription complex PAF1C to stop the production of IFN-stimulated genes (ISGs) (Shah et al., 2018).

Besides, the functionality of IFN machinery can be affected by DENV through few mechanisms. For example, the non-structural NS2B/3 are able to downregulate the antiviral responses by interrupting the IFNα/β induction pathways (Aguirre et al., 2012) while the other non-structural proteins such as NS2A, NS4A and NS4B can affect the IFN signaling among cells by causing partial blockage on the STAT signaling pathway (Muñoz-Jordán et al., 2005).

### 2.3 Cross reaction within DENV serotypes and among different flaviviruses

The NS1 protein for all DENV serotypes share the similar sequence up to 70% and its sequence also shows 40–50% similarities with other flaviviruses (Xu et al., 2016). Therefore, the cross reaction among DENV of different serotypes and DENV with other flaviviruses will always happen. Based on the concurrent infection of DENV-1 and DENV-2 happened in Brazil (dos Santos et al., 2003) and concurrent infection of DENV-2 and DENV-3 detected in Somalia (Kanesa-thasan et al., 1994), Taiwan (Wang et al., 2003) and China (Wenming et al., 2005), Araújo, F. M. and his team shown that *Aedes aegypti* might possibly infected by combinations of different arboviruses or they might have the capability to carry out simultaneous arboviruses transmission (Araújo et al., 2006). As assumed by Wenming et al. (2005), the transmission of concurrent infection of DENV-2 and DENV-3 by infected mosquitoes might occur at places with the presence of more than one type of serotypes (Wenming et al., 2005). Besides, there are also co-infections between DENV and ZIKV (Dupont-Rouzeyrol et al., 2015) and between DENV and Chikungunya virus (CHIKV) (Caron et al., 2012) being reported.

Similar with DENV, Zika virus (ZIKV) is also a positive single stranded RNA virus which originated from *Flaviviridae* family (Pierson and Diamond, 2013). Zika virus infection normally shows non-specific clinical signs such as conjunctivitis, mild fever, headache and rash which can easily cause confusion with other flaviviruses infection like dengue and chikungunya (Musso et al., 2015). Besides, ZIKV infection can also affect pregnant women by causing fetal malformations and birth defects to babies such as microcephaly (Abrahamsson et al., 2016). A travel-related case which was initially detected as dengue virus infection due to false positive result from the NS1 antigen test, was then suspected to be acute zika virus infection (Gyurech et al., 2016).

When ZIKV is causing a secondary infection to a patient who is previously been infected by another flavivirus infection such as DENV, this person is likely to have DENV background immunity in his body and give a positive result for DENV detection which leads to cross-reactivity in the IgM test. However, the cross-reactivity in the IgM test will be low if the ZIKV is the primary infection (Lanciotti et al., 2008). As reported by George et al. (2017), a patient who previously been infected by ZIKV will have higher primary DENV-2 viremia followed by changes correlated with serious DENV infection (George et al., 2017). This is due to the high concentration of DENV cross-reactive antibodies triggered by the ZIKV infection which have low neutralization effect towards DENV-2 (Lanciotti et al., 2008) and leads to non-neutralizing concentrations of DENV-2 cross-reactive antibody responses. These responses also increased the chances of getting heterologous serotypes infection from either DENV-1, 3 or 4 which implies that pre-existing immune responses towards ZIKV might induced the ADE of infection from all DENV serotypes (George et al., 2017).

### 2.4 Mechanism and effects of ADE

The first type of ADE mechanism is known as intrinsic ADE where internalization of DENV immune complexes will increase the “burst size” of infected cells by suppressing the intracellular antiviral response (Flipse et al., 2016; Halstead, 2014). DENV can enter the host cell through phagocytosis by forming DENV-antibody complex with the sub-neutralizing antibodies and enter the cell through antibody-mediated phagocytosis (Ayala-Nunez et al., 2016; Halstead et al., 2010; Narayan and Tripathi, 2020). When 2 macrophage Fcγ receptors ligate and induce interleukin-10 (IL-10) production, it will lead to bias T-helper-2 (Th2) response (Chareonsirisuthigul et al., 2007; Ubol et al., 2010) due to inhibition of RIG-I/MDA5 and JAK-STAT pathways (Ubol et al., 2010) which cause the release of type 1 interferon, interleukin 12, interferon γ and TNF become downregulated (Halstead et al., 2010). In short, intrinsic ADE modifies the innate immune system and intracellular mechanism to enhance the viral replication (Halstead et al., 2010).

**Fig. 3:**
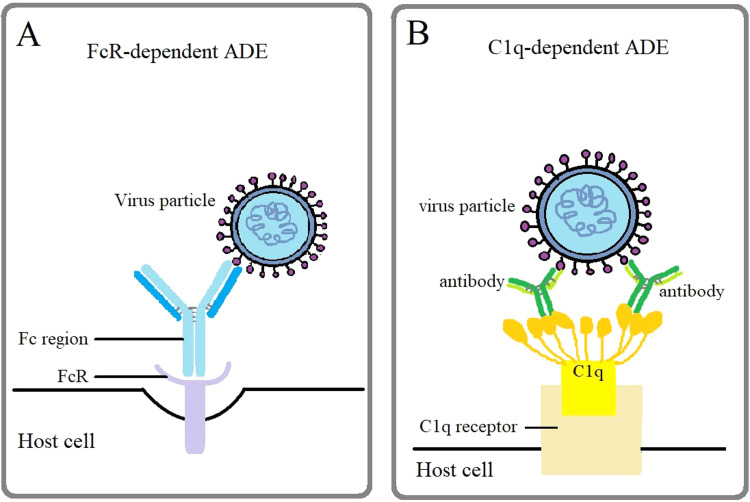
Different mechanisms of antibody dependent enhancement (ADE). Viral entry can be facilitated through different pathways with the help of (A) Fc receptors or (B) C1q receptors to be endocytosed into the cell membrane and further causing ADE.

Another type of ADE mechanism is the extrinsic ADE. Two common types of ADE mechanisms are Fc receptor (FcR)-dependent ADE and C1q-dependent ADE (Fig. 3). FcR-dependent ADE is the most common mechanism for a wide range of virus infection such as HIV, influenza A, dengue and Ebola. Cells which have FcR such as monocytes, B cells, neutrophils and macrophages are easily attached by the virus-antibody complexes because the Fc region of the antibody will bind on the cell surface with FcR which leads to increment in virus attachment to cell surfaces (Hawkes, 1964). Once the DENV-immune complexes repress the antiviral immune response which is responsible for the production of interleukin-12 (IL-12), interferon gamma (IFN-γ), TNF-α and nitric oxide radicals (NO), the IL-6 and IL-10 expression will be activated and stimulate the propagation of virus particle which resulted in the increase in infected cells and viral particles followed by the happening of ADE (Chareonsirisuthigul et al., 2007).

C1q-dependent ADE mechanism is the initiation of complement classical pathway which is prevalent in Ebola virus (EBOV) infection (Takada et al., 2007). In this mechanism, the ADE is facilitated by the complement protein C1q and C1q receptor (C1qR) (Takada et al., 2003; Takada and Kawaoka, 2003) which was distinguished in majority of the mammalian cells (Eggleton et al., 1998; Nicholson-Weller and Klickstein, 1999) even though the FcRs are fully expressed in immune system which comprised of B cells, neutrophils and monocytes or macrophages (Takada and Kawaoka, 2003).

When a virus binds to the non-neutralizing antibodies or antibodies at sub-neutralizing level, this will ease the viral entry and further enhances the viral infection which could be the secondary infection. This condition refers to antibody dependent enhancement (ADE) (Taylor et al., 2015). Initially, the pre-existing antibodies can neutralize homologous DENV infection by blocking the binding between DENV and natural receptor on a cell surface but it will allow a heterologous dengue virus to enter and travel through the primary infection pathway which leads to ADE due to high virus burden (Tirado and Yoon, 2003).

Cross reactive antibodies are produced through the stimulation of immunological memory after years of infection to neutralize more than one type of DENV serotypes (Beltramello et al., 2010). These antibodies normally have poor neutralizing activity and tend to attach with immunodominant epitopes with low availability which resulting in higher rates of ADE and cross reactivity (Beltramello et al., 2010; Halstead and O'Rourke, 1977). As reported by Mongkolsapaya et al., the existence of heterologous memory and cross-reactive CD4^+^T cells will worsen the infection during immune-recall responses for secondary infection (Mongkolsapaya et al., 2003).

Non-neutralizing antibodies induce by antibody response during the hetero-serotype secondary infection may cause intrinsic ADE (Wilder-Smith et al., 2019) which may lead to a more serious dengue infection (Narayan and Tripathi, 2020). This type of ADE can greatly enhance the viral replication at early stage of infection compared to extrinsic ADE (Narayan and Tripathi, 2020). This is because of its ability to escape from innate immune response and migrate freely without being easily detected by endogenous interferon pathway due to the antibody-mediated internalization (Flipse et al., 2016).

## 3 Therapeutic agents for DENV infection

### 3.1 Neutralizing antibodies and their mechanism

Neutralizing antibodies play important role in inhibiting the functional site of DENV envelope (E) protein from interacting with the host cells (Pierson et al., 2008). The antibodies which are highly neutralizing normally bind to the epitopes on E protein which are readily accessible and exposed on the virion surface. However, non-neutralizing antibodies including neutralizing antibodies at sub-neutralizing level might lead to ADE when they bind to DENV particle (Pierson et al., 2007; Rey et al., 2018).

Taking the neutralization mechanism in West Nile Virus as example, the mechanism was known as a “multiple hit” model (Pierson et al., 2007), which is also similar with the neutralization in other viruses using the virion “coating” by antibodies model (Burton et al., 2001). The neutralization effect is dependent on the concentration of neutralizing antibody. For a potential neutralizing antibody to achieve the minimum stoichiometry of neutralization, approximately 30 antibodies were needed to occupy a virus particle. The factors which might affect the interaction between neutralizing antibody and viral epitope includes the accessibility of viral epitopes, antibody concentration and binding affinity (Pierson et al., 2007).

The infectivity of a virus particle coated by neutralizing antibodies can be blocked in different ways, one of it is the crosslinking of virus particles through the Fab arms of antibody in order to form a large cross-linked virus/antibody aggregates which can stop the spreading of virus particles by lowering the concentration of virus particles which can infect cells (Chan et al., 2011). Other than cross-linking, the inhibition of E protein conformational change and receptor binding can avoid membrane fusion between virus particle and endosomal membranes which allow the release of viral genome into cytoplasm (Rey et al., 2018). Some of the neutralizing antibodies might have dual activity which can block the membrane fusion and at the same time interrupt the receptor binding mechanism, leading to blocking of viral entry through endosome. When the neutralization titres of neutralizing antibodies in pre-attachment assay formats exceeded the neutralization titres in post-attachment (Crill and Roehrig, 2001; Deng et al., 2011; Hasan et al., 2017), overall neutralization might occur and allow double level protection against DENV particle (Rey et al., 2018).

Regarding the problem of neutralization escape, this can be settled by utilization of potential neutralizing mAbs cocktails which were specific towards different epitopes to block the mutant escape from neutralization. The research of Wang et al. (2017) shown that the recombinant tetravalent symmetric antibody engineered with LALA mutation was very reactive in inhibiting ZIKV infection in a mouse model by neutralizing the escape mutants which were individually developed from each parent antibodies and the growth of escaped mutants were inhibited (Wang et al., 2017).

### 3.2 Potential neutralizing antibodies for DENV infection

The E protein of DENV is always the major concern for developing neutralizing antibody (Roehrig, 2003). Each ectodomains of DENV E proteins consists of three distinctive domains such as domain I (EDI), II (EDII) and III (EDIII) which join them together to exist as dimers (Modis et al., 2003; Rey et al., 1995; Zhang et al., 2004). Normally, neutralizing epitopes located at places such as the hinge area within EDI, end of EDII and EDIII lateral surface (Modis et al., 2005; Oliphant et al., 2005; Pierson et al., 2008; Rey et al., 1995). Among the matched pairs of neutralizing mouse monoclonal antibodies (mAbs) and three types of domains E protein, the epitopes and A strand from EDIII was recognized by most of the strongly neutralizing mAbs (Shrestha et al., 2010). The exposed quaternary binding sites on matured virions were targeted by potential neutralizing antibodies via crosslinking with the E dimers on the virions’ surface, resulting in inhibition of conformational changes, thus stopping the formation of fusion membrane (Fibriansah et al., 2015; Zhang et al., 2016).

1A1D-2 is a neutralizing antibody which can prevent the conformational change of E protein and formation of membrane fusion by blocking at the EDIII position (Volk et al., 2007). Gandham and his team developed thioaptamers (DENTA-1) to target on the EDIII by utilizing the neutralizing mechanism of neutralizing antibody 1A1D-2 because of its excellent binding properties (Yang et al., 2006). Since the filter binding assays indicated that the thioaptamers able to bind tightly on the DENV-2 EDIII, DENTA-1 has high possibility to exhibit the same neutralizing effect as 1A1D-2. For a matured virion under static state, the binding region of neutralizing antibody and EDIII is located at the non-surface exposed β−1 strand which revealed that the unexposed regions can be easily in contact with solvent and ease the neutralizing activity of antibody by targeting to the binding region (Lok et al., 2008). Another research done by Deng and her team also proved that antibody 2B8 which is specific to EDIII binding is capable in inhibiting the attachment of DENV serotype 2 in BHK cells (Deng et al., 2011).

The discovery of broadly neutralizing antibodies which has the potential in neutralizing all DENV serotypes is reported by Dejnirattisai and the team (Dejnirattisai et al., 2015). They found that mAbs which target on the envelope dimer epitope (EDE) manage to neutralize DENV from infected human cells and insect cells efficiently compared to antibodies which target on fusion loop epitope (FLE). One of the reasons might be due to the capability of anti-EDE antibodies which can recognize the DENV at different maturation stages with different prM concentrations (Dejnirattisai et al., 2015), unlike anti-FLE antibodies which are very dependent on the presence of prM for fusion loop binding (Cherrier et al., 2009).

Besides that, the discovery of engineered antibody such as bispecific antibody is also one of the popular approaches in developing therapeutic antibody against DENV. The combination of two mAbs with specific functionalities to generate bispecific antibody (DVD-1A1D-2A10) has been discovered to retain and exhibit the parental activities (Kou et al., 2010). This bispecific antibody manage to interrupt the virus attachment and fusion processes during early infection of DENV (Li et al., 2008) because it consists of 1A1D parental antibody which is able to interact with EDIII to inhibit the attachment of DENV on host cell and another parental antibody 2A10 which bind with EDII domain to interfere the fusion process between the viral particles and endosomal membrane (Deng et al., 2011; Nybakken et al., 2005). With the advantages of dual blocking functionalities, the blocking of viral attachment and fusion are possible to happen simultaneously or either one of the processes can be inhibited, the authors predict that the inhibition of DENV infection can be more effective. DENV E protein possess the ability to alter its structure into dimer and trimer form during virus attachment and fusion process, respectively (Li et al., 2008), however the bispecific antibody has the benefit of avoiding the changes of E protein structure due to its parental activity which allows it to bind to both EDII and EDIII domain simultaneously (Shi et al., 2016). Besides that, this bispecific antibody also reveals complete neutralizing activity at the concentration of 1.33 µM comparing with its individual parental antibodies which only show 70% neutralization effect at the same concentration (Shi et al., 2016). The generation of bispecific antibody is quite promising to be develop as a therapeutic antibody to fight against DENV.

Previously, a team of researchers had developed human monoclonal antibody which possessed great cross neutralization against all DENV serotypes (Setthapramote et al., 2012) but showed ADE activities when tested against DENV-2 (Sasaki et al., 2013). In their recent study, they constructed DNA plasmids containing heavy or light chain genes from previous human monoclonal antibody to express 1G7C2_hG1 antibodies. Besides, they also modify Fc CH2 domain region of the plasmid carrying heavy chain gene and perform another expression of 1G7C2_hG1-LALA antibodies. The expressed antibodies are specific against all DENV serotypes. For post-transfection analysis, these antibodies are detectable in 2 days after transfection and their concentrations raise up till it reach the maximum level on Day 6. For the cross-neutralizing activity, both 1G7C2_hG1 and 1G7C2_hG1-LALA antibodies neutralize 90% of DENV-1, 100% neutralization against DENV-2 and 98% to 100% neutralization against DENV-3 and DENV-4. The analysis of ADE activities on 1G7C2_hG1 shows that it can induce ADE but the degree of antibody-enhanced infection is lower than its parental antibody. On the other hand, 1G7C2_hG1-LALA demonstrates great neutralizing effect without enhancing activities against all serotypes, which indicates that the modification of Fc CH2 domain region can eliminate enhancing activity. The secretion of this antibody in BALB/c mice is generated within 3 days after immunization and achieve a maximum amount of about 1300 ng/mL on Day 5 and 7. In general, the 1G7C2_hG1-LALA antibody can provide great neutralization effect without enhancing activity to all DENV serotypes (Benjathummarak et al., 2021).

### 3.3 Antiviral peptides

As one of the antiviral agents, peptide inhibitors aim to interrupt with the DENV life cycle by affecting the functions of viral proteins (Songprakhon et al., 2020). Antiviral peptides can be used to inhibit DENV infection through a few routes such as (i) recognizing the host cellular receptors to block the entry of virus by avoiding the host cell from the viral proteins attachment, (ii) targeting of structural proteins which will prevent the viral entry by blocking the binding between host cells and virus, (iii) targeting of non- structural proteins which mostly deal with the inhibition of viral replication (Chew et al., 2017). However, there are some drawbacks for the antiviral peptides to be used as efficient therapeutic drug such as the weak stability and limited bioavailability (Diao and Meibohm, 2013). One of the methods used to solve these issues is by applying chemical modifications to alter the physiochemical properties of the peptides (Gentilucci et al., 2010), another method is improving the antiviral peptides properties through mutagenesis assays (Schmidt et al., 2010).

Some antiviral peptide target on dengue protease such as NS2B-NS3 which involves in viral polyprotein cleavage to release structural and non-structural proteins upon maturation (Perera and Kuhn, 2008). Ltc 1 exhibit better inhibiting ability towards the protease at high temperature (40 °C) and lowers the viral load in infected cells. Hence, it is possible that Ltc 1 manage to disturb the life cycle of DENV when it inhibits the activity of NS2B-NS3 protease (Rothan et al., 2014). There are also other inhibitors against NS2B-NS3 protease which has the potential to be developed as antiviral agents (da Silva-Júnior and de Araújo-Júnior, 2019; Dražić et al., 2020; Rothan et al., 2013; Takagi et al., 2017).

Hrobowski and the team suggested that viral entry and fusion can be blocked by using peptide which can mimic the highly conserved regions of class II fusion proteins (Hrobowski et al., 2005). They identified a peptide inhibitor DN59 which mimic stem domain of DENV E protein is capable in inhibiting DENV-2 infection at low concentration. It exhibits highest inhibitory activity of 100 ± 0.5% at 20 µM and it's IC50 is recorded at approximately 10 µM. It also showed excellent specificity towards DENV-2 infection with more than 99% of inhibitory activity against plaque formation at low concentration. Besides than having inhibitory activity against DENV-2, this peptide inhibitor also exhibits inhibitory effect against West Nile virus (WNV) due to their similar conserved regions, thus it may be useful as broad-spectrum peptide inhibitors for flaviviruses (Hrobowski et al., 2005). Further research was carried out and the researchers determined that this peptide inhibitor had inhibitory effect against all DENV serotypes with the IC50 at 2 to 5 µM. Interestingly, they also found out that DN59 manage to disrupt viral membrane and release viral genome. The genome was totally separated from the E protein after treated with DN59 as it forms holes on the viral membrane due to its strong interaction with liposome vesicles and disrupt the lipid bilayers, thus causing the virus to loss its infectability (Hollmann et al., 2021; Lok et al., 2012).

Besides, the mimicking capability of MLH40 peptide allows it to interrupt the DENV M-E interactions by mimicking the helical amphipathic stem region and conserved hydrophobic loop of M protein ectodomain which causes the alteration of homodimer E protein structure when it binds to DENV and thus inhibits the viral infection for all types of DENV (Panya et al., 2015).

The interaction between DENV C protein and lipid droplets (LDs) is important for the formation of dengue viral particles (Samsa et al., 2009). Using the principle of molecular mimicry, peptide can inhibit the formation of DENV particle by mimicking the disordered N-terminal region of C protein including NML + R motif which is believed to be the interaction site of LD-C protein (Martins et al., 2012). There is another finding related to antiviral mechanism involving DENV C protein (Xia et al., 2020). A compound inhibitor ST148 which shows potential in inhibiting DENV replication (Byrd et al., 2013) reveals its “kissing” interaction with two capsid dimers, forming an inhibitor-bound capsid tetramer which could happen before or during virus assembly. Virions which contain this compound inhibitor will have faulty effect during nucleocapsid uncoating when infecting new cells. This will further lead to the degradation of C protein and RNA genome. Thus, this mechanism might be a useful insight for future antiviral approach (Xia et al., 2020).

On the other hand, Kaptein and co-researchers identified a promising and potent pan-serotype DENV inhibitor known as JNJ-A07. It is an inhibitor targeting on NS3 and NS4B interaction. The formation of NS3-NS4B complex is one of the important parts for viral replication (Chatel-Chaix et al., 2015), therefore the inhibitor interrupts with the complex formation by causing conformational changes at the cytosolic loop. Their study showed that the inhibition of interaction between NS3 and NS4B demonstrated a promising antiviral mechanism with great inhibitory effect. This inhibitor has good pharmacokinetic and it shows promising inhibitory activities against different 21 combinations of genotypes and serotypes (Kaptein et al., 2021).

### 3.4 Vaccine development and potential vaccine candidates for DENV infection

The development of vaccines had been a great challenge in dengue therapeutics due to the complication of its four antigenically distinct serotypes which can cause infection. A primary infection from one of the DENV serotype will result in long term homotypic protection but with a short term heterotypic protection against infections from other serotypes, thus a person might face disease enhancement during a second heterotypic infection (Halstead, 1970). The production of long-term antibody-secreting plasma cells through the formation of germinal centers (GC) in secondary lymphoid tissues with the aid of follicular helper T cells (Tfh) is important for developing an effective dengue vaccine (Havenar-Daughton et al., 2020).

Until now, the only authorized vaccine available in worldwide is known as Dengvaxia (CYD-TDV) (Thomas and Yoon, 2019) which is a live chimeric, attenuated and tetravalent vaccine consisting a non-structural Yellow fever 17D strain virus backbone with combination of structural pre-membrane (prM) and envelope (E) genes of the four DENV serotypes (Guy et al., 2015). However, Dengvaxia is only applicable for infected person who are between 9 and 16 years old (Hadinegoro et al., 2015).

Other vaccine candidates are still on the way of succeeding through clinical trials of different stages form Phase I until Phase III. The vaccine candidates which are undergoing phase III clinical trials include TV003/TV005 (NCT01506570) and TDV/DENVax/TAK003 (NCT02302066) (Sáez-Llorens et al., 2017; Whitehead et al., 2017). Vaccine candidate TDEN-LAV (NCT01702857) (Dorigatti et al., 2015) is still undergoing phase II clinical trials, TDEN-PIV (NCT01666652) (Guy et al., 2015) and D1ME100/TVDV (NCT00290147) (Beckett et al., 2011) are currently in the stage of phase I clinical trial while V180 (DEN-80E) (NCT01477580) had finished phase I clinical trial (Manoff et al., 2015).

Graham and her team had recently developed a new live attenuated tetravalent vaccine (DLAV) which induce rapid production of DENV specific-multifunctional T cells in 8 to 14 days after getting vaccinated and retained for at least 6 months. Besides, DLAV also induce effector memory T cells re-expressing CD45RA (T_EMRA_) which maintained its elevated frequency for 1 year of post-vaccination. This suggests that the immunity protection induce by this vaccine is very promising (Graham et al., 2020).

In previous study, Pinto's group demonstrated that both E protein-based DNA vaccine (pE1D2) (Azevedo et al., 2011) and NS1-derived DNA vaccine (pcTPANS1) (Costa et al., 2006) manage to induce immune protection in immunized mice model (Pinto et al., 2019). In their recent study, they are more interested to find out the protective immunity which can be induced by the combination of 2 DNA vaccine candidates. Their study resulted in excellent immunity protection in mice vaccinated with pE1D2+pcTPANS1 DNA vaccines due to the combined immune response. Even after the lethal challenge with DENV-2, there is no morbidity found in the pE1D2+pcTPANS1 immunized mice and none of them showed clinical sign of infection due to the reinforcement of immune response (Pinto et al., 2022).

All potential therapeutic agents discussed above is summarised in Table 1.

**Table 1:** Summary of potential therapeutic agents with respective DENV serotypes and binding target.

Therapeutic agents	DENV serotypes	Target	Highlights	Refs.
Peptide inhibitor	All	DENV NS1	Unique binding sites of NS1 targeted by peptides.	(Songprakhon et al., 2020)
Synthetic peptide	DENV2	NS1	Generation of synthetic peptide with unique epitope which is immunogenic towards antipeptide antibody in rabbits as a potential serotypic specific detection tools.	(Guevarra et al., 2020)
Peptide inhibitor	DENV1–4	DENV E proteins	MLH40 peptide (24–31 µM) manage to inhibit all serotypes and 80% inhibition effect against DENV2 was achieved at 100 µM.	(Panya et al., 2015)
Peptide inhibitor	All	stem domain of DENV E proteins	DN59 possesses great inhibitory activity to all DENV serotypes at low concentration. It also able to disrupt viral membrane and release viral genome.	(Hrobowski et al., 2005; Lok et al., 2012)
Neutralizing antibody	DENV-1	CC’ loop of DIII	Neutralizing activity of antibody which targets on cryptic epitopes can be affected by genotypic variation of DENV.	(Austin et al., 2012)
Broadly neutralizing antibody	All	EDE	Monoclonal antibodies which bind to the EDE shows broadly neutralizing effect for all DENV serotypes.	(Dejnirattisai et al., 2015)
Neutralizing human monoclonal antibody	All	N/A	Utilization of gene modification in DNA plasmids to express 1G7C2_hG1-LALA antibodies which can provide cross neutralization effect to all DENV serotypes without ADE activity.	(Benjathummarak et al., 2021)
Bispecific antibody	All	EDII, EDIII domain	This bispecific antibody maintains the binding abilities from its parental antibodies and possess better neutralizing effect against DENV.	(Shi et al., 2016)
Vaccine candidate	DENV	Modified NS1 wing domain	Generation of mAb 33D2 which possess both in-vitro and in-vivo inhibition.	(Lai et al., 2017)
Vaccine candidate	DENV	Modified NS1	Do not cross react with platelets or uninfected endothelial cells and the prolonged bleeding time induced by DENV is reduced significantly using dengue haemorrhagic mouse model	(Wan et al., 2014)
Vaccine candidate	DENV2	E protein and NS1	Combination of E protein-based and NS1-derived DNA vaccines provide potent immunity protection in immunized mice model.	(Pinto et al., 2022)
Antiviral agent	All	C protein	A small molecule ST-148 had been discovered to have inhibition reaction on the replication of four serotypes of DENV	(Byk and Gamarnik, 2016)
Antiviral agent	DENV2	C protein	Virions assembled with inhibitor-bound capsid tetramers cannot uncoats the nucleocapsid well and resulting in DENV replication inhibition.	(Xia et al., 2020)
Virus inhibitor	Different serotypes and genotypes	NS3-NS4B complex	JNJ-A07 shows promising inhibitory activities against different 21 combinations of genotypes and serotypes by blocking the NS3 and NS4B interaction.	(Kaptein et al., 2021)
Anti-dengue therapeutic	–	NS2B-NS3 protease	Latarcin peptide (Ltc 1) significantly inhibits the spreading and replication of virus by interacting with NS2B-NS3 protease	(Rothan et al., 2014)
Neutralizing antibody	DENV2	EDIII	mAb DB32–6 is the clone with strongest neutralizing activity, it was further converted to humanized antibody which preserved its original neutralizing effect against different strains of DENV2.	(Li et al., 2012)

## 4 DENV diagnosis

Sensors which provide quantitative assessment with high specificity are important for better monitoring and early detection (Mustapa et al., 2018) in Dengue diagnosis. One of the common assays used for DENV diagnosis includes Enzyme-Linked Immunosorbent assay (Alteri et al., 2020) (Alcon et al., 2002). This assays has few limitations such as expensive testing kit and required few hours for the detection of targeted interest (Huy et al., 2011). Other than that, the utilization of virus isolation to diagnose DENV infection during acute stage is time consuming and required very skilful while handling the cell culture or animal models (Yamada et al., 2002). For reverse transcription-polymerase chain reaction (RT-PCR) method, it is able to detect early DENV infection at febrile stage but also time-consuming with complicated procedures (Ahmed and Broor, 2014). The immunochromatographic assays also commonly used for DENV detection especially the assay which applied gold nanoparticles conjugated antibodies as capture protein (Tanaka et al., 2006). Gold nanoparticles (AuNPs) is a promising nanomaterials applied in lateral flow assay due to advantages such as stable, nontoxic (Amina and Guo, 2020; Daniel and Astruc, 2004), biocompatibility and ease of functionalization with many biomolecules (Pissuwan et al., 2020).

The detection of NS1 has been applied in many types of monoclonal or polyclonal antibody-based immunoassays due to the high level of NS1 antigen in infected patient's sera which allows it to be a promising biomarker in many commercial types of immunoassays such as lateral flow rapid assays and ELISA kits (Antunes et al., 2015; Gowri Sankar et al., 2012; Pal et al., 2014, 2015; Ranzoni et al., 2015; Sánchez-Purrà et al., 2017). The circulation of viral antigens will remain longer time in the bloodstream of dengue infected patients compared to viral RNA, thus the detection of NS1 antigen is more preferrable when the viral RNA is undetectable in RT-PCR (Koraka et al., 2003).

**Fig. 4:**
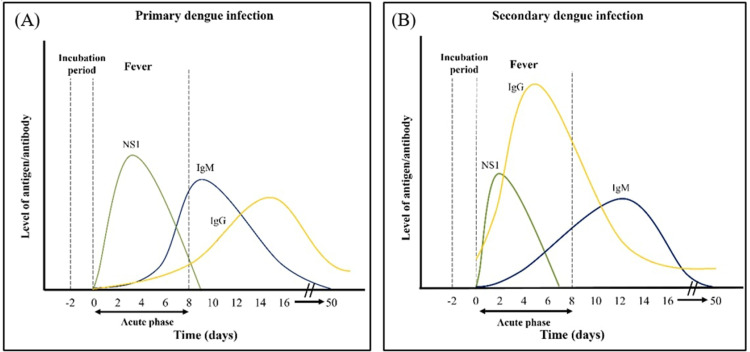
The level of NS1, IgM and IgG for primary and secondary dengue infection. (A) During primary infection, NS1 and IgM are detectable starting day 0 onwards and day 3 onwards, respectively, while IgG can only be detected on day 10 onwards. (B) For secondary infection, IgG level is used to differentiate both primary and secondary infection by presenting very significant level during acute phase due to rapid anamnestic IgG reaction.

The level of NS1 antigen, IgM and IgG during primary and secondary infection is illustrated in Fig. 4. During primary infection, NS1 antigen can be detected at the early infection starting from day 0 onwards and a slight peak will be obtained in day 4. The antigen detection would not be affected by the existence of IgM even though NS1 and IgM will be present simultaneously starting from day 3 to day 9 of infection. On the other hand, the detection of IgM can only be performed after 3 to 4 days of onset and it will achieve persisted amount in the serum from day 5 onwards (Alcon et al., 2002). Effective detection of IgG can only be done starting from day 10 to 15 after the onset of symptoms. Normally, IgG detection assays will be used to differentiate primary and secondary infection depending on the IgG level. This is because IgG will only raise to a significant level after a few weeks or months of primary infection (Peeling et al., 2010). After onset of symptoms during secondary infection, IgG is detectable on day 3 onwards due to its quick anamnestic response which resulted in high level of IgG in early stage (Muller et al., 2017).

### 4.1 Current commercial diagnostic kits

The detection of NS1 can be an effective way for the early diagnosis of DENV infection once the fever onset begins (Chuansumrit et al., 2008; Lapphra et al., 2008) due to its appearance in the early stage (Paranavitane et al., 2014), which is within Day 0 to Day 9 (Dussart et al., 2006; Shu et al., 2002) and maximum level can be detected within Day 6 to 10 (Xu et al., 2006). In the study of Alcon et al., starting from the first day of symptoms’ onset, NS1 antigen was found to be circulating in the sera up to 9 days and the concentration of NS1 increased from 0.04 lb/mL on Day 0 to 2.00 lb/mL on Day 7 in the sera tested from acute phase serum samples while the sera concentration of convalescent stage starting from Day 8 onwards was 0.04 lg/mL. For secondary infection, NS1 in haemolymph concentration was around 0.01 to 2 lb/mL and it was not detected in the sera samples of convalescent stage (Alcon et al., 2002). Therefore, most of the commercial kits focus on the detection of NS1 as the biomarker for DENV diagnosis due to its detectable significant amount which is available in early stage.

According to the evaluation done by S. D. Blacksell and his team, the sensitivity and specificity of commercial NS1 antigen point-of-care diagnosis tests ranging from 48.5% to 58.6% and 92.5% to 99.4%, respectively (Blacksell et al., 2011). Based on the comparison on the sensitivity and specificity of different commercial NS1 diagnostic tests done by Osorio L. team, SD BIOLINE™ NS1/IgM/IgG assay provides the highest detection sensitivity up to 80.7% followed by SD BIOLINE™ NS1/IgM (78.4%) and second generation Pan E™ (71.1%) (Osorio et al., 2010). There are other studies which also conducted review on different types of commercial diagnostic kits (Guzman et al., 2010; Hunsperger et al., 2009)

The commercial SD Bioline Dengue Duo test is the common diagnosis assay which consist of 2 types of evaluation test for the detection of DENV NS1 and anti-DENV IgG or IgM in the serum of patients (Andries et al., 2012). Its benefit is the additional evaluation on the detection of DENV IgM and IgG which increase the detection sensitivity which is complementary for NS1 detection assay. The improved sensitivity of SD Duo test is again proven by a study by comparing the combined detection of NS1/IgG/IgM and the detection of each individual biomarker. The NS1/IgG/IgM test shown the highest sensitivity (90.65%), followed by IgG test (90.06%), NS1 test (87.50%) and lastly IgM (60.515) (Sánchez-Vargas et al., 2014). The combination of two or more dengue biomarkers such as NS1 with IgM or NS1 with IgM and IgG seem to have improving effect on the overall performance of diagnostic kits from 53.5% to 88.7% (Wang and Sekaran, 2010) and from 60.61% to 90.65% (Sánchez-Vargas et al., 2014), respectively.

However, the individual positive result from either IgG or IgM detection can also indicate infection from past few months which can only be considered as presumptive diagnosis (Tricou et al., 2010). Moreover, the IgM test has been reported to have low sensitivity (Hunsperger et al., 2009; Vickers et al., 2015) when using it as a single biomarker where the negative results obtained from IgM test cannot completely resolve the suspects of dengue infection (Vickers et al., 2015), therefore IgM test is more suitable to be used to detect recent infection but not as a marker for acute infection diagnosis (Blacksell, 2012).

### 4.2 Limitations of current diagnosis tests

In the study done by Jayathilaka and her team, majority of the antibody which detect specifically on NS1 was directed towards the highly conserved NS1 regions, which resulted in cross reaction with JEV and WNV for more than 65%. The production of NS1 antibodies could be due to infection from other flaviviruses and further increased to a higher antibody titre during the secondary infection by DENV (Jayathilaka et al., 2018). In addition, the false positivity in a RDTs is usually due to the presence of residual antibodies from previous infections by any type of flaviviruses or different dengue serotypes which frequently happen in endemic areas (Blacksell et al., 2011).

Chung mentioned that the commercial diagnostic kits seem to have high variability ranging from 37% to 98.9% which is possibly due to the decrease in sensitivity with the increase in time especially after the fever onset during secondary infections from viral serotype 2, serotype 4 when a considerable amount of DENV-reactive IgG is found in the serum (Chuansumrit et al., 2008; Hang et al., 2009; Koraka et al., 2003; Lapphra et al., 2008) which is proven in the study of Osorio et al. (2010) where the lowered detection sensitivity happened in cases from secondary infections with the onset of fever for 4 days and severe infections (Osorio et al., 2010). Relating with the time-dependent sensitivity of diagnostic kits, the duration of illness is said to be affecting the sensitivity of diagnostic kits because the study of Guzman team reported that the kits are more sensitive when tested against acute sera samples of Asian patients which were collected during first 3 to 4 days of illness compared to sera samples collected in later phase (Guzman et al., 2010).

Besides that, the sensitivity of diagnostic kits also differs according to different types of DENV serotypes. In the study of Guzman et al. (2010), the sensitivity of Platelia kit against DENV-2 was the lowest compared to other serotypes and the differences in sensitivity of diagnostic kits against different serotypes might be related to the specific NS1 epitope of different serotypes and different origin of the same serotypes (Guzman et al., 2010).

Despite of all the availability of commercial DENV Diagnostic kits, there are still some existing limitations as listed in Table 2.

**Table 2:** Limitations of commercial DENV diagnostic kits.

Assay Kit	Target	Country	Serotypes	Limitations	Refs.
Platelia™ dengue NS1 Ag kit	NS1 detection	Brazil	DENV4	Low sensitivity, delay in detection	(Sea et al., 2013)
Panbio® Dengue Early ELISA	NS1	Brazil	DENV4	False negative results	(Colombo et al., 2013)
SD Bioline Dengue Duo test	NS1 test	Santos, Brazil	DENV2, DENV4	False negative, low sensitivity in secondary dengue infection	(Andries et al., 2012; Chuansumrit et al., 2008; Colombo et al., 2013; Felix et al., 2012; Pok et al., 2010)
SD Bioline Dengue Duo test	IgM	Puerto Rico, Malaysia	DENV	Low sensitivity in acute and secondary infection	(Hunsperger et al., 2009)

### 4.3 Improvements of DENV diagnosis and discovery of promising diagnostic markers

The study of Nascimento et al. (2018) found out that anti-dengue NS1 IgG and IgG3 have the potential to be developed as biomarkers for long term DENV infection instead of the common biomarkers used to detect acute infection such as IgA and IgM antibodies (Balmaseda et al., 2003). This is possibly due to their consistent presence in the first week of infection for early detection after the onset of DENV infection symptoms and higher detection window which is within 4 to 6 months after the onset of symptoms (Nascimento et al., 2018). In contrast, NS1 antigen detection has limited detection time within first few days of onset to maximize the detection sensitivity (Ahmed and Broor, 2014).

Bio-functionalized tapered optical fiber is applicable as a label-free quantitative diagnosis tool in DENV diagnosis due to rapid detection time, simple process, portable usability and easy fabrication. The presence of IgG antibody will be higher during early onset of secondary infection compared to primary infection, therefore provides a higher possibility for the capture of antibody by the antigen bio-receptor. The work of Mustapa and his team focused on the detection of anti-NS1 IgG antibody by immobilizing the DENV NS1 antigen on the tapered multimode fiber (TMMF). They determined that the bio-functionalized TMMF can detect the antibody at concentration of 100 pg/mL within 5 min and its sensitivity can be up to 7 × 10^−6^ a.u/pg/mL (Mustapa et al., 2018). This bio-functionalized TMMF could be utilized in DENV diagnosis for better sensitivity and rapid diagnosis result.

Mustapa and his team's recent study is on improving the biosensing performance of TOF by incorporation of nanomaterials such as polyamidoamine (PAMAM) dendrimer. It is a hyper-branched macromolecules which can act as an active layer when it is integrated into TOF. This strategy may increase the active sites of the TOF sensor for target antigen attachment, mainly due to the polar functionalities of the PAMAM dendrimer which is able to anchor the protein at their edges and improve protein loading (Mustapha Kamil et al., 2019; Satija et al., 2014). Their study integrated the PAMAM dendrimer into TOF sensor as active layer to enhance antibody absorption so that more active sites are available for DENV-2 E proteins binding during immobilization. This improvised PAMAM integrated TOF sensor showed sensing affinity with K_d_ value of 1.02 × 10^−10^ M, which is better compared to their previous study (Kamil et al., 2018). This might be owing to the utilization of PAMAM which offer more binding sites and provide larger surface area for the E protein attachment (Mustapha Kamil et al., 2019). Their study proven that the utilization of PAMAM dendrimer as active layer in bio-functionalized TOF sensor greatly enhance its performance in recognizing DENV E proteins.

Previously, many works have been conducted on utilizing serotype-specific antibodies in DENV diagnostic assay but these approaches did not work well in differentiating all DENV serotypes (Cabezas et al., 2008; Ding et al., 2011; Qiu et al., 2009). Instead of using antibodies produced from hybridoma technology, the serotype-specific antibodies derived from human naïve phage display libraries reported to show multiplexed diagnosis by binding with single pan-reactive capture antibody due to their specific epitopes (Lebani et al., 2017). These epitopes were distinctive from the wing domain epitope which is one of the most accessible epitopes in NS1 (Akey et al., 2014). Hence, the application of serotype-specific antibodies derived from human naïve phage display libraries could be giving an idea in resolving the challenges of DENV serotypes differentiation.

A researcher managed to generate hybridoma antibodies which can detect all DENV serotypes and ZIKV without cross reaction from mice immunized with recombinant DENV1, DENV2, DENV3, DENV4 and recombinant ZIKV protein. Effective detection is only applicable to serum samples collected during acute phase because this is the phase where both NS1 and RNA are detectable. The detection limit of hybridoma antibodies for serotype-specific test and pan-DENV test ranging from 1 to 20 ng/mL while 20 ng/mL is the detection limit for ZIKV NS1 detection (Bosch et al., 2017).

A serum samples which contain high concentration of IgG may causes the NS1 antigen to form immune complexes with the IgG and unable to be detected, which further affects the sensitivity of diagnostic kits (Hang et al., 2009). The high level of IgG is normally present in the serum samples from patients with secondary dengue infections (Andries et al., 2012). For some cases of blood-borne viruses such as Hepatitis B and C, the dissociated antibody-antigen immune complexes play important role in the early stage of diagnosis (Panakitsuwan et al., 1997; Troisi and Hollinger, 1997). According to Koraka and his team, the immune complexes form by NS1 antigen and IgG needs to be broken down to detect the presence of NS1 in the blood serum of patients with secondary infection (Koraka et al., 2003).

The research on virus-targeting sdAb is getting popular and they discovered that sdAb can be a promising tool in both therapeutic and diagnostic areas (Fernandes et al., 2017; Hultberg et al., 2011; Sherwood and Hayhurst, 2013; Wu et al., 2017). Nanobodies refer to antigen binding fragments with the smallest molecular size which is approximately 15 kDa (Cortez-Retamozo et al., 2004). It can be derived from sharks and members of the Camelidae family such as llama and camelid which do not have light chains (Hacisuleyman and Erman, 2020). One of the examples of nanobody is single domain antibody (sdAb) which comprised of complementarity determining regions (CDRs) such as CDR1, CDR2 and CDR3. The CDR3 is an unusual loop which is long and extended to penetrate and bind to the cleft of antigen, allowing it to have high affinity in target binding (Hacisuleyman and Erman, 2020; Wesolowski et al., 2009). The sdAbs are highly thermostability up to 95 °C and manage to retain its binding affinity due to its ability to refold during thermal denaturation (Flajnik et al., 2011; Griffiths et al., 2013; Hussack et al., 2011; Liu et al., 2007). The thermostability of sdAb can be one of the solutions for the problem faced in rapid diagnostic tests using conventional mAb (Griffiths et al., 2013).

The human single chain variable antibody fragments (HuScFv) which has specific binding affinity towards the R and E domains of NS1, was proven to be effective in reducing viral replication in host cell infected with influenza virus when it was exposed with HuScFv. This is probably due to its interference towards intracellular NS1 production and restoration of the host innate immune activity (Yodsheewan et al., 2013). It is known that anti-NS1 antibody with Fc region will somehow induce the complement-mediated cytolysis which might worsen the DENV infection (Lin et al., 2003). Hence, the first antibody development which focus on completely HuScFv against NS1 is reported. This HuScFv does not consists of Fc region and it is much smaller compared to conventional IgG. The ability of HuScFv to bind with NS1 in both native and secreted form suggests that it might be able to interrupt the roles of NS1. This study demonstrated that the virus replication process can be interrupted through the interaction between the HuScFv and NS1 due to the reduction in virus release (Poungpair et al., 2014).

With comparisons between commercial kit SD Bioline Dengue Duo, mAb immobilized kit and VHH antibody immobilized kit, VHH immobilized kit achieved the highest specificity (99.50%) among the 3 diagnostic kits and its detection limit for recombinant NS1 is 4.5 ng/mL, which is much lower than the mAb immobilized kit (9 ng/mL). The detection limit obtained from VHH antibody immobilized kit is not only falls within the range of detection limit for circulating NS1 during acute stage (10 ng/mL to 50 µg/mL) (Alcon et al., 2002), but it is much lower than that indicating this kit is significantly sensitive to detect low level of DENV2 NS1 (Fatima et al., 2014). Fatima and her team also use epitope mapping to identify the potential epitope for both the VHH antibody and mAb. They discover that all the binding peptides from both types have homologous region corresponding to amino acid sequence from positions 224 to 232 of the NS1 antigen. The His and Trp appeared in almost every clone suggesting that these two amino acids are essential for antibody binding, especially His residue (Fatima et al., 2014; Wu et al., 2001, 2003). This VHH antibody immobilized kit is a very potential diagnostic tools along with its advantages in efficiency, sensitivity, specificity and binding affinity compared with mAb immobilized kit.

Genetic engineering modification can be applied on sdAb to improve the detection limits of immunoassays. The utilization of sdAb as capture protein has its limitation during the covalent interaction with the bigger total surface area of complex matrices. Its small molecular size will have difficulties in binding ability towards extensive binding interface which will further affect the detection of target protein and assay sensitivity (Trilling et al., 2013, 2014). An interesting approach was demonstrated by constructing protein engineering on the sdAb to obtain a SpyTag fusion sdAb in its dimer form which can form SpyCatcher/SpyTag pair by immobilizing on the SpyCatcher coated microspheres. The application of SpyCatcher/SpyTag pair helps to increase significant detection signal for NS1 from human serum spiked with all DENV serotypes compared with the non-oriented sdAb. The increment of detection limits up to 5-fold is the promising result for utilization of SpyTag fusion sdAb in DENV diagnosis and other protein capture application (Anderson et al., 2019).

The enlightening part of each subject discussed above is summarised in Table 3.

**Table 3:** Highlights of summarised improvements of DENV diagnosis and discovery of promising diagnostic markers.

Improvements on diagnosis/discovery of diagnostic markers	DENV serotypes	Target	Highlights	Refs.
Bio-functionalized TMMF	–	Anti-NS1 IgG antibody	Bio-functionalized TMMF with immobilized NS1 can detect the antibody at concentration of 100 pg/mL within 5 min.	(Mustapa et al., 2018)
PAMAM integrated TOF sensor	DENV-2	E protein	Improvised bio-functionalized TOF sensor has sensing affinity which is better due to the utilization of PAMAM dendrimer which offer more binding sites and provide larger surface area for the E protein attachment.	(Mustapha Kamil et al., 2019)
Hybridoma antibodies	All	N/A	These hybridoma antibodies can detect all serotypes without cross-reaction.	(Bosch et al., 2017)
HuScFv	DENV2	NS1	As specific anti-DENV biomolecules.	(Poungpair et al., 2014)
Ilama derived sdAb	–	NS1	The selected pair of sdAbs show the best specificity against NS1 for all DENV serotypes, do not cross react with ZIKV, YFV, TBEV but show some binding with JEV and WNV.	(Shriver-Lake et al., 2018)
Ilama derived VHH antibody	DENV2	NS1	Rapid diagnostic kit which utilized VHH antibody as capture antibody is more specific and sensitive compared to conventional mAb due to its long CDR3 region which can reach to the hindered antigen cleft.	(Fatima et al., 2014)
SpyTag fusion sdAb	All	NS1	The application of SpyCatcher/SpyTag pair helps to increase significant detection signal for NS1 from human serum spiked with all DENV serotypes.	(Anderson et al., 2019)

## 5 Conclusion

The widespread of DENV is already an existing public health issue for a long time, with the outbreak of COVID-19 pandemic, some Asia countries will need to face double burden especially the impacts on economic and health sector (Harapan et al., 2021). The more concerning part is majority of the COVID-19 cases ranged from mild to moderate illnesses with similar symptoms commonly happen in dengue infection such as fever and rash (Henrina et al., 2020), thus there is high possibility that COVID-19 cases and dengue cases will be misdiagnosed or a person will be having dual infection from both virus (Masyeni et al., 2021) just like DENV and other flaviviruses. Besides, there are cases of cross reaction been reported in countries like Indonesia, Thailand and Singapore (Kembuan, 2020; Prasitsirikul et al., 2020; Yan et al., 2020).

Up to now, the issue of cross reaction due to infection by different DENV serotypes and different flaviviruses are still not resolved yet. In addition, there is also higher possibility for ADE condition to happen and worsen the disease condition. Apart from these problems, the habit of DENV to escape from immune response is also one of the considerations to be taken in controlling the DENV infection effectively.

To solve the issue of cross reaction, many approaches have been carried out unceasingly to discover useful and potential antiviral drugs, neutralizing antibodies and vaccine candidates which can act efficiently and effectively. The approaches include application of genetic engineering, nanobodies derivation, molecular mimicry, discoveries of antigenic cryptic epitopes, structural study and inhibition mechanism. Also, the development of a more sensitive and specific diagnosis alternative is very in need to solve the limitations of current commercial diagnostic kits so that the rapid and early diagnosis for flaviviruses or other emerging virulent viruses can be achieved for immediate clinical management on those infected patients. Approaches such as nanobodies derivatives and antibody engineering are interesting idea in developing sensitive and specific biomarker detectors. Interestingly, NS1 could possibly develop as a potential biomarker for DENV detection because many biomarker detectors can target on NS1 antigen specifically as mentioned above. In conclusion, the development for both therapeutic and diagnostic aspects are providing useful and interesting ideas for future development.

## Declaration of Competing Interest

The authors declare that they have no known competing financial interests or personal relationships that could have appeared to influence the work reported in this paper.

## Data Availability

Data will be made available on request.
